# Recombinant Human KAI1/CD82 Attenuates Glucocorticoid-Induced Muscle Atrophy by Promoting Myogenic Differentiation

**DOI:** 10.3390/ijms27062555

**Published:** 2026-03-11

**Authors:** Dong Hwan Kim, Hyesook Lee, Jung-Hwa Han, Yun Jeong Kang, Roo Gam Jeong, Jin Hur, Hyun Sik Gong

**Affiliations:** 1 Department of Orthopedic Surgery, Sindorim Seoul Orthopedic Clinic, Seoul 08210, Republic of Korea; rayman09.k@gmail.com; 2 Department of Convergence Medicine, Pusan National University School of Medicine, Yangsan 50612, Republic of Korea; lhyes0219@gmail.com (H.L.); hjh2735@pusan.ac.kr (J.-H.H.); jeong0531@gmail.com (Y.J.K.); esworld88@naver.com (R.G.J.); 3 R&D Center, Simplycarebio Co., Ltd., Room 607, 88 Somang-gil, Juchon-myeon, Gimhae-si 06125, Republic of Korea; 4 Research Institute for Convergence of Biomedical Science and Technology, Pusan National University Yangsan Hospital, Yangsan 50612, Republic of Korea; 5 PNU GRAND Convergence Medical Science Education Research Center, Pusan National University School of Medicine, Yangsan 50612, Republic of Korea; 6 Department of Orthopedic Surgery, Seoul National University Bundang Hospital, Seongnam-si 13620, Republic of Korea

**Keywords:** recombinant human KAI1 (*rh*KAI1), muscle atrophy, myogenic differentiation, dexamethasone

## Abstract

Sarcopenia and glucocorticoid-induced myopathy are significant forms of muscle atrophy that pose considerable public health challenges. In this regard, preventing muscle atrophy is crucial for enhancing quality of life and increasing life expectancy. In this study, we investigated the effect of recombinant human KAI1 (*rh*KAI1) on myogenic differentiation and its protective effect against dexamethasone-induced muscle atrophy. *rh*KAI1 enhanced myogenic differentiation in both murine C2C12 myoblasts and primary human endometrial stromal cells, as evidenced by upregulation of myogenic regulatory factors and increased myotube formation. These effects were accompanied by increased phosphorylation of Akt and AMPK. In a dexamethasone (Dex)-induced atrophy model, *rh*KAI1 increased myotube diameter, restored MyHC expression, and reduced the expression of the E3 ligase atrogin-1, accompanied by increased phosphorylation of Akt and AMPK. In addition, *rh*KAI1 administration improved Dex-induced functional impairment in mice, as reflected by increased grip strength and improved rotarod performance. Molecular analyses further showed that *rh*KAI1 modulated Dex-induced fiber-type-related gene expression by restoring MYH7 (type I) and reducing MYH4 (type IIb) expression. Collectively, our findings demonstrate that *rh*KAI1 promotes myogenic differentiation and alleviates several functional and molecular features associated with glucocorticoid-induced muscle deterioration. These results support the potential of *rh*KAI1 as a candidate molecule for further investigation in steroid-induced muscle dysfunction.

## 1. Introduction

Skeletal muscle, constituting over 40% and 30% of total body mass in men and women, respectively, is essential for numerous vital functions, including posture maintenance, respiration, whole-body metabolism, glucose homeostasis, and injury repair [1,2]. Consequently, preserving muscle health and developing effective therapies to prevent or treat muscle atrophy are crucial for overall health and longevity [3]. Muscle atrophy, characterized by a decrease in muscle mass and function resulting from an imbalance between protein synthesis and degradation [4], arises from diverse etiologies including inactivity, aging, hormonal imbalances, injury, nerve damage, and various pathological conditions [5,6]. Muscle atrophy significantly impairs motor function, contributing to locomotive syndrome and increasing the risk of falls, fractures, and subsequent disability [7]. Conditions such as sarcopenia and glucocorticoid-induced myopathy are prominent examples of muscle atrophy, posing significant public health challenges [8]. Sarcopenia, prevalent among the elderly, and glucocorticoid-induced muscle wasting, often a side effect of treatments such as dexamethasone, are both associated with increased fall risk, functional decline, hospitalization rates, and mortality [9,10]. This reduction in muscle mass and strength diminishes quality of life, leading to exercise intolerance and difficulties with activities of daily living [11]. Ultimately, severe muscle atrophy is associated with increased morbidity and mortality [12,13]. Despite extensive research, effective pharmacological treatments remain elusive, with existing therapies limited by side effects or inadequate efficacy [14,15,16]. Current clinical management often focuses on non-steroidal anti-inflammatory drugs (NSAIDs) for pain relief, but these are associated with significant adverse effects [17,18]. This unmet clinical need highlights the urgent need for novel therapeutic strategies to combat muscle atrophy. The development of effective treatments is further hampered by an incomplete understanding of the underlying pathophysiological mechanisms driving muscle atrophy.

KAI1, also known as CD82, belongs to the tetraspanin superfamily, a group of transmembrane proteins characterized by four transmembrane helices, short intracellular N- and C-termini, and two extracellular loops [19,20]. This structural architecture enables KAI1 to organize dynamic protein complexes within the cell membrane, influencing a broad array of cellular functions [21,22]. Although KAI1 is expressed in multiple tissue types, its expression levels vary considerably depending on the tissue context. In addition, KAI1 has been identified as a cell surface marker in specific stem cell populations [19]. Differentially expressed KAI1 is closely associated with malignant tumors and serves as a potential biomarker [23]. Previous studies have established that KAI1 has diverse roles across various biological contexts. Initially recognized for its metastasis-suppressive effects, KAI1 inhibits cancer cell motility and invasiveness [24,25]. Beyond its anti-cancer effects, KAI1 also functions as an endogenous inhibitor of angiogenesis, modulating blood vessel formation through growth factor regulation via the Src/p53 pathway [19]. In addition, we reported that human recombinant KAI1 (*rh*KAI1) protein attenuates transforming growth factor (TGF)-β1-mediated epithelial–mesenchymal transition (EMT) in retinal pigment epithelial cells via Smad-dependent signaling [26]. More recently, we demonstrated that *rh*KAI1 exhibits regulatory effects on M1 macrophage polarization, suggesting anti-inflammatory activity. Furthermore, several studies have explored the multifaceted roles of tetraspanin KAI1 and its implications for muscle health. Fontelonga et al. [20] reported that KAI1 interacts with dysferlin and myoferlin, crucial for membrane repair; its deficiency exacerbates dystrophy, increasing muscle weakness, and fibrosis. They focused on the interaction of KAI1 with dysferlin and myoferlin in muscle cells, suggesting its involvement in membrane repair. They found that KAI1 deficiency in dystrophic mice worsened disease symptoms, suggesting an important role for KAI1 in muscle health. Hall et al. [27] further explored the role of KAI1 in muscle stem cell activation and overall muscle function. They demonstrated that KAI1 deficiency impaired muscle stem cell activation, and reduced muscle repair efficiency. This led to a worsened dystrophic phenotype in mice, emphasizing KAI1’s essential role in maintaining healthy muscle function. Both studies highlight the potential of KAI1 as a therapeutic target for muscular dystrophy, although further research is needed to fully elucidate its mechanisms of action. Despite these established roles, the therapeutic potential of KAI1 for muscle regeneration and the treatment of muscular dystrophy remains largely unexplored. Therefore, this study aims to assess the myogenic differentiation capacity of *rh*KAI1 protein and elucidate its mechanism of action in a dexamethasone-induced model of muscle atrophy. We will evaluate its therapeutic efficacy and delineate its underlying molecular mechanisms within this experimental system.

## 2. Results

### 2.1. rhKAI1 Promotes Myoblast Differentiation into Myotubes

**Figure 1:**
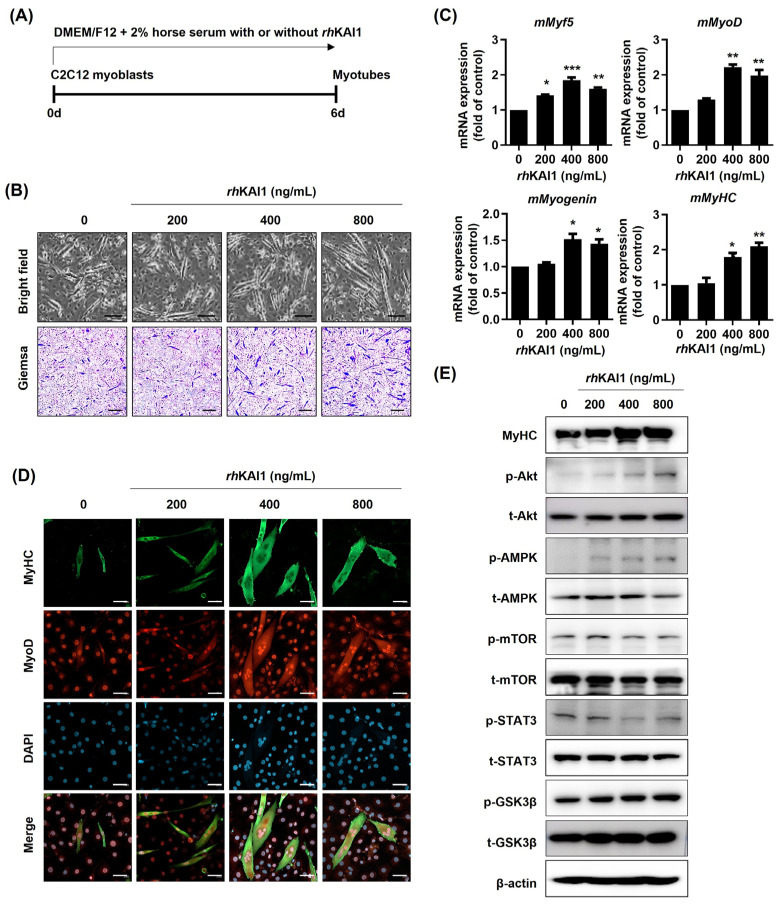
*rh*KAI1 enhances C2C12 myoblast differentiation into myotubes. C2C12 myoblasts were cultured in DMEM/F12 supplemented with 2% horse serum, with or without *rh*KAI1 (0, 200, 400, and 800 ng/mL) for 6 days. (**A**) Schematic representation of the experimental timeline. (**B**) Morphological assessment of C2C12 cells treated with different concentrations of *rh*KAI1. Bright-field and Giemsa-stained images are shown. Scale bar: 100 μm. (**C**) mRNA expression levels of myogenic regulatory factors (mMyf5, mMyoD, mMyogenin, and mMyHC) in C2C12 cells treated with *rh*KAI1. Data represent the mean ± SD. * *p* < 0.05, ** *p* < 0.01, *** *p* < 0.001 vs. control. (**D**) Immunofluorescence staining of differentiated C2C12 cells for MyHC (green) and MyoD (red), with DAPI (blue) for nuclear staining. Scale bar: 50 μm. (**E**) Western blot analysis of myogenic signaling molecule expression in C2C12 cells treated with *rh*KAI1. Total (t-) and phosphorylated (p-) proteins are shown. β-actin served as a loading control.

To investigate the effect of *rh*KAI1 on myoblast differentiation, C2C12 cells were cultured in a differentiation medium containing various concentrations of *rh*KAI1 (0, 200, 400, and 800 ng/mL) for 6 days. Morphological analysis revealed a dose-dependent increase in myotube formation, evident in both bright-field and Giemsa-stained images (Figure 1B). Specifically, treatment with 400 ng/mL and 800 ng/mL *rh*KAI1 resulted in significantly elongated and more numerous myotubes compared to the control group. This enhanced myogenesis was further confirmed by qRT-PCR analysis, which showed a statistically significant upregulation of myogenic regulatory factor mRNA expression mMyf5, mMyoD, mMyogenin, and mMyHC (Figure 1C). As shown in Figure 1D, immunofluorescence staining also corroborated these findings, showing increased MyHC and MyoD protein expression in cells treated with higher concentrations of *rh*KAI1, consistent with the increased myotube formation observed in Figure 1B. Western blot analysis was performed to examine changes in key signaling molecules involved in myogenesis (Figure 1E). The results showed increased phosphorylation of Akt and AMPK in *rh*KAI1-treated cells. However, the phosphorylation and expression levels of mTOR, STAT3, and GSK3β remained largely unchanged. Taken together, these results show that *rh*KAI1 promotes myoblast differentiation into myotubes in C2C12 cells in a dose-dependent manner and is accompanied by increased phosphorylation of Akt and AMPK.

### 2.2. rhKAI1 Induces Myogenic Differentiation of Primary Human Endometrial Stem Cells

**Figure 2:**
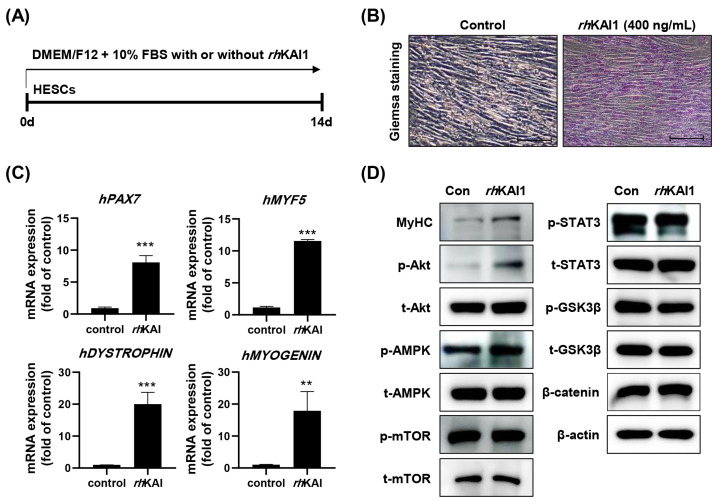
*rh*KAI1 induces myogenic differentiation in primary human endometrial stromal cells (HESCs). HESCs were treated with 400 ng/mL *rh*KAI1 for 14 days. (**A**) Schematic representation of the experimental design. (**B**) Giemsa staining of HESCs; scale bar: 200 μm. (**C**) mRNA expression levels of myogenic regulatory factors (hPAX7, hMYF5, hDYSTROPHIN, and hMYOGENIN) in *rh*KAI1-treated HESCs, relative to untreated controls. Data are presented as mean ± SD; ** *p* < 0.01, *** *p* < 0.001 vs. control. (**D**) Western blot analysis of proteins involved in myogenic signaling pathways in *rh*KAI1-treated HESCs. Total (t-) and phosphorylated (p-) forms of proteins are shown; β-actin served as a loading control.

To investigate the effect of *rh*KAI1 on the myogenic differentiation potential of primary human endometrial stem cells (HESCs), cells were cultured with 400 ng/mL *rh*KAI1 for 14 days. Giemsa staining showed a significant increase in the formation of multinucleated myotubes in the *rh*KAI1-treated group compared to the control, indicating enhanced myogenic differentiation (Figure 2B). This observation was further supported by qRT-PCR analysis, which revealed a significant upregulation of hPAX7, hMYF5, hDYSTROPHIN, and hMYOGENIN mRNA expression in *rh*KAI1-treated HESCs (Figure 2C). These results strongly suggest that *rh*KAI1 promotes myogenic differentiation in HESCs. Western blot analysis was performed to examine changes in signaling molecules associated with myogenic differentiation (Figure 2D). The results showed increased phosphorylation of Akt (p-Akt) and AMPK (p-AMPK) in the *rh*KAI1-treated group. These findings indicate that *rh*KAI1-induced myogenic differentiation of HESCs is accompanied by increased phosphorylation of Akt and AMPK.

### 2.3. rhKAI1 Protects C2C12 Myotubes Against Dexamethasone-Induced Atrophy

**Figure 3:**
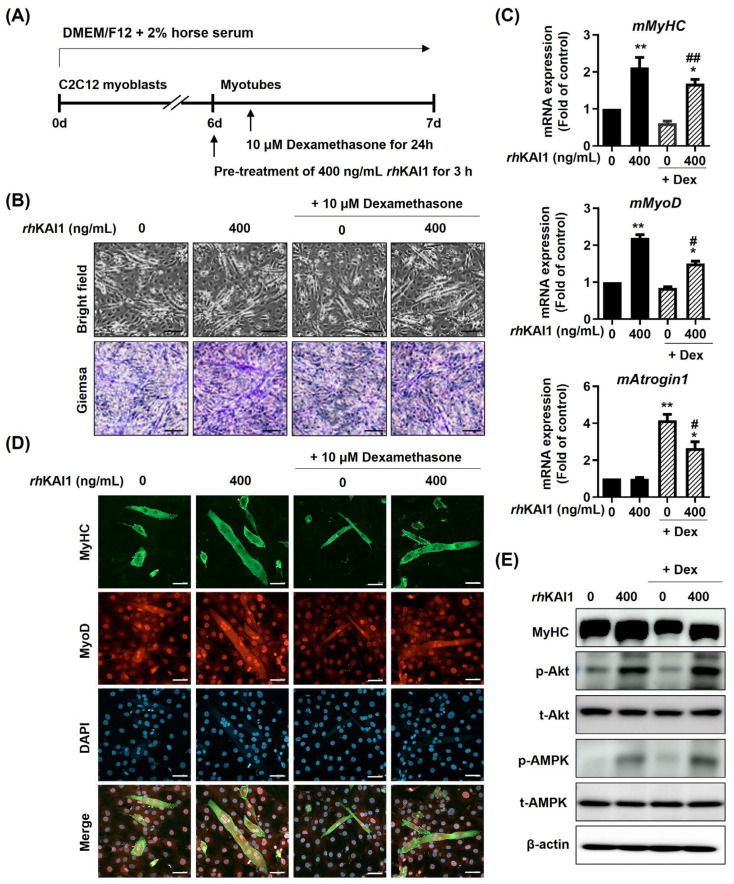
*rh*KAI1 protects C2C12 myotubes from dexamethasone-induced atrophy. C2C12 myotubes were pretreated with 400 ng/mL *rh*KAI1 for 3 h prior to treatment with 10 μM dexamethasone (Dex) for 24 h. (**A**) Schematic diagram of the experimental design. (**B**) Morphological analysis of C2C12 myotubes: (top) Bright-field microscopy; (bottom) Giemsa staining; scale bar: 100 μm. (**C**) mRNA expression levels of myogenic markers (mMyHC, mMyoD) and an atrophy-related gene-1 (mAtrogin-1) in C2C12 myotubes. Data represent the mean ± SD; * *p* < 0.05, ** *p* < 0.01 vs. control; # *p* < 0.05, ## *p* < 0.01 vs. Dex-treated cells. (**D**) Immunofluorescence staining of C2C12 myotubes for MyHC (green) and MyoD (red); nuclei were counterstained with DAPI (blue); scale bar: 50 μm. (**E**) Western blot analysis of proteins involved in myogenic signaling pathways in C2C12 myotubes.

To investigate the protective effects of *rh*KAI1 against dexamethasone-induced muscle atrophy, C2C12 myotubes were pretreated with 400 ng/mL *rh*KAI1 for 3 h before exposure to 10 μM dexamethasone (Dex) for 24 h. Dexamethasone treatment alone caused a significant reduction in myotube size and density, as observed by bright-field and Giemsa staining (Figure 3B). This morphological atrophy was accompanied by significant molecular changes. While Dex treatment alone did not significantly affect mMyHC or mMyoD mRNA levels, *rh*KAI1 pretreatment significantly increased their expression (Figure 3C). Importantly, Dex treatment induced a significant increase in mAtrogin-1 mRNA, a marker of muscle atrophy; this increase was significantly attenuated by *rh*KAI1 pretreatment. Immunofluorescence staining confirmed these findings, showing reduced MyHC and MyoD protein expression with Dex treatment alone, an effect that was rescued by *rh*KAI1 pretreatment (Figure 3D). Western blot analysis was performed to examine changes in signaling molecules associated with Dex-induced muscle atrophy. Figure 3E shows that Dex treatment significantly reduced the phosphorylation of AMPK (p-AMPK) and Akt (p-Akt) in C2C12 myotubes. However, *rh*KAI1 pretreatment significantly restored p-AMPK and p-Akt levels. The restoration of p-AMPK and p-Akt was observed in parallel with the protective effects of *rh*KAI1 against Dex-induced myotube atrophy.

### 2.4. rhKAI1 Ameliorates Dexamethasone-Mediated Muscle Atrophy in Mice

**Figure 4:**
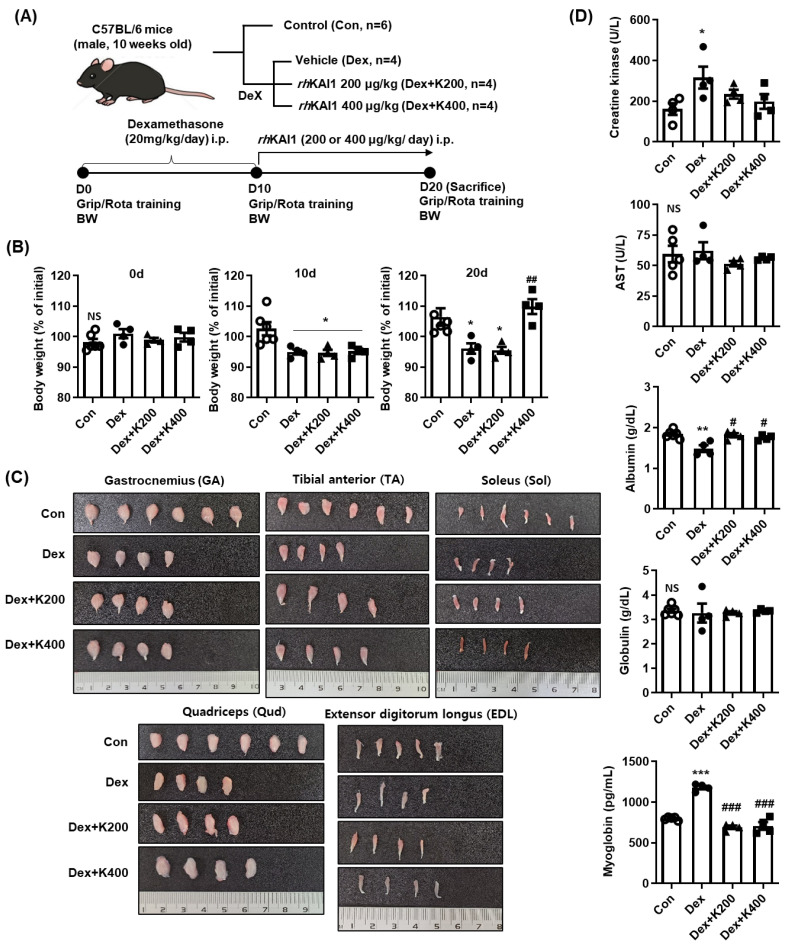
Effects of *rh*KAI1 on body weight, skeletal muscle mass, and serum biochemistry in dexamethasone-induced muscle atrophy. C57BL/6 mice received daily intraperitoneal (i.p.) injections of 20 mg/kg dexamethasone for 10 days to induce muscle atrophy. After 10 days of Dex treatment, mice were treated with i.p. injections of normal saline (Dex group) or *rh*KAI1 (200 or 400 µg/kg body weight) for 10 days. Control mice received normal saline injections for 20 days. (**A**) Schematic representation of the experimental timeline and assessments. (**B**) Percent change in body weight relative to baseline at days 0, 10, and 20. Data are expressed as mean ± SD. NS, not significant; * *p* < 0.05 vs. control; ## *p* < 0.01 vs. Dex group. (**C**) Representative images of isolated gastrocnemius (GA), tibialis anterior (TA), soleus (Sol), quadriceps (Qud), and extensor digitorum longus from the hind limbs. (**D**) Serum levels of creatine kinase (CK), aspartate aminotransferase (AST), albumin, globulin, and myoglobin. Data are expressed as mean ± SD. NS, not significant; * *p* < 0.05, ** *p* < 0.01, *** *p* < 0.001 vs. control; # *p* < 0.05, ## *p* < 0.01, ### *p* < 0.001 vs. Dex group.

A mouse model of muscle atrophy was induced by administering dexamethasone daily for 10 days. The effect of *rh*KAI1 administration on Dex-induced muscle alterations was then evaluated. During the study period, body weight changes were monitored. A significant decrease in body weight was observed in the Dex-treated group compared to baseline (Figure 4B). Among the animals treated with *rh*KAI1 for a subsequent 10 days, the group receiving 400 μg/kg *rh*KAI1 showed a partial recovery of body weight compared with the Dex-treated group. As shown in Figure 4C, no significant differences were observed in the weights of isolated skeletal muscles, including gastrocnemius (GA), tibialis anterior (TA), soleus (Sol), quadriceps (Qud), and extensor digitorum longus (EDL), among the groups. Serum creatinine, aspartate aminotransferase (AST), and globulin levels showed no significant differences among the groups (Figure 4D). However, serum albumin levels were significantly decreased in the Dex-treated group compared to the control group, while *rh*KAI1 treatment restored albumin levels to control levels. Furthermore, serum myoglobin levels were approximately 1.3-fold higher in the Dex-treated group (1090.7 pg/mL) than in the control group (811.4 pg/mL). *rh*KAI1 treatment reduced this increase, bringing myoglobin levels close to those observed in the control group.

**Figure 5:**
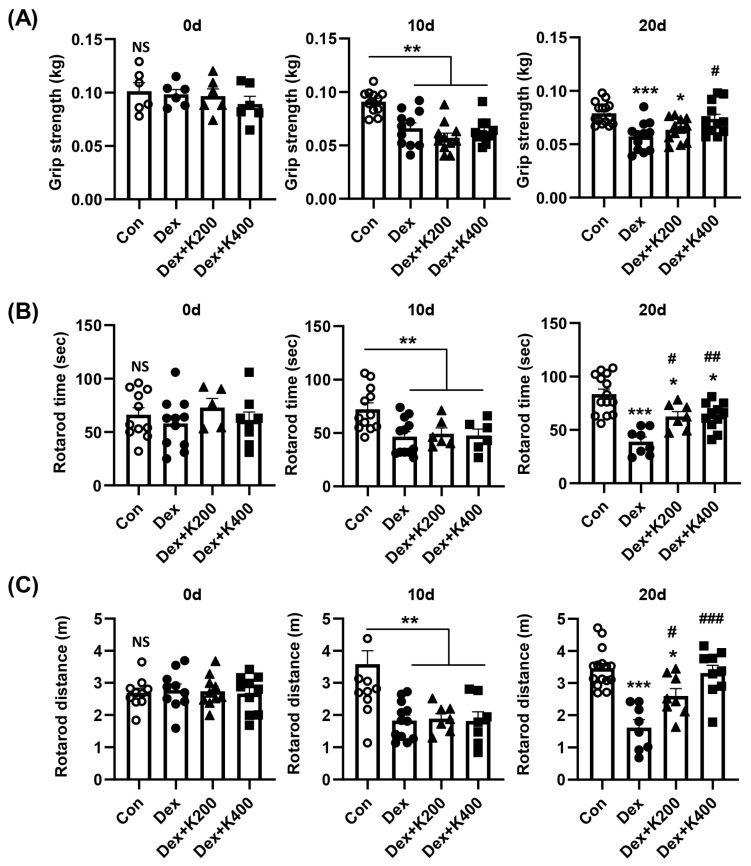
*rh*KAI1 improves skeletal muscle function in dexamethasone-induced atrophic mice. (**A**) Forearm grip strength of experimental animals at 0, 10, and 20 days. This test was repeated three times, and the average value was recorded for each mouse. (**B**,**C**) Motor coordination was measured at 40 rpm for 180 s, and each mouse underwent three rotarod trials. (**B**) Duration on the rotarod; (**C**) Distance traveled on the rotarod. Data are expressed as mean ± SD. NS, not significant; * *p* < 0.05, ** *p* < 0.01, and *** *p* < 0.001 vs. control group; and # *p* < 0.05, ## *p* < 0.01, ### *p* < 0.001 vs. Dex group.

To assess muscle function, grip strength and rotarod tests were performed at days 0, 10, and 20. Dexamethasone treatment resulted in a significant decrease in grip strength compared to the control group at days 10 and 20 (Figure 5A). *rh*KAI1 treatment showed a dose-dependent improvement in grip strength, particularly at day 20 (Figure 5A). Similarly, the rotarod test, assessing motor coordination and endurance, revealed a significant decrease in both the time spent on the rotarod and the total distance traveled in the DEX-treated group at days 10 and 20 compared to the control group (Figure 5B,C). Treatment with *rh*KAI1 (400 µg/kg) significantly improved both rotarod time and distance compared with the Dex-treated group at day 20.

**Figure 6:**
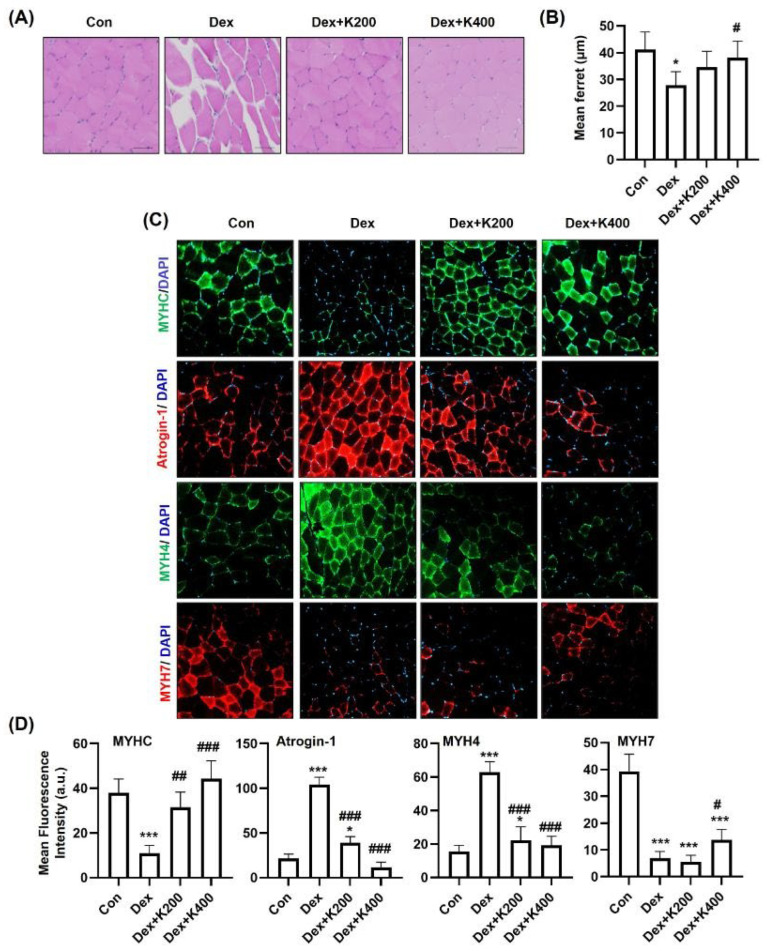
*rh*KAI1 modulates muscle regeneration in dexamethasone-induced atrophic mice. (**A**) Representative histological images of GA muscle sections stained with H&E. Scale bar: 200 μm. (**B**) Quantification of myofiber size expressed as minimal Feret’s diameter (~90 fibers per group). (**C**) Immunofluorescence staining of GA muscle sections for muscle fiber type characterization. MyHC (green), Atrogin-1 (red); MyH4 (type IIB, green); MyH7 (type I, red); DAPI (nuclei, blue). Scale bar: 50 μm. (**D**) Quantification of mean fluorescence intensity (MFI) using ImageJ version 1.53. Data are expressed as mean ± SD. * *p* < 0.05, and *** *p* < 0.001 vs. control group; and # *p* < 0.05, ## *p* < 0.01, ### *p* < 0.001 vs. Dex group.

To further investigate the effects of *rh*KAI1 on muscle tissue, histomorphological analysis of the GA muscle was performed. Representative H&E-stained images (Figure 6A) showed a marked reduction in myofiber size in the dexamethasone-treated group compared with the control group, which was partially attenuated by *rh*KAI1 treatment. Quantitative analysis of myofiber size using minimal Feret diameter confirmed a significant decrease in the Dex group, which was partially restored in the *rh*KAI1-treated groups (Figure 6B). Immunofluorescence staining (Figure 6C) revealed decreased MYHC expression and markedly increased Atrogin-1 expression in the Dex group, consistent with muscle atrophy. In addition, Dex treatment increased MYH4 (type IIB fiber) expression while decreasing MYH7 (type I fiber) expression. Quantification of fluorescence intensity further confirmed that *rh*KAI1 treatment partially reversed these changes in MYHC, Atrogin-1, MYH4, and MYH7 expression (Figure 6D), suggesting an improvement in muscle fiber integrity and fiber-type-related markers. Taken together, these findings indicate that *rh*KAI1 treatment improved body weight recovery, grip strength, rotarod performance, and histological features in a mouse model of Dex-induced muscle atrophy.

## 3. Discussion

Skeletal muscle regeneration is a highly coordinated process that involves the activation, proliferation, and differentiation of muscle stem cells, known as satellite cells [28,29]. Upon injury or physiological stress, quiescent satellite cells are activated and express early myogenic transcription factors such as Pax7 and MyoD, followed by induction of downstream regulators including Myf5, Myogenin, and MyHC, which drive terminal myogenic differentiation and myotube formation [30,31]. C2C12 cells, a murine myoblast line derived from satellite cells, have been extensively utilized as a surrogate model for skeletal muscle regeneration due to their robust ability to undergo fusion and differentiation under appropriate stimuli [32,33]. The formation of mature myotubes through myogenic differentiation is not only crucial for muscle development but also essential for effective regeneration following muscle atrophy [34,35]. Impaired differentiation capacity contributes to defective regenerative responses seen in aging and catabolic conditions such as sarcopenia or glucocorticoid-induced muscle atrophy [36,37]. Thus, facilitating myogenesis through exogenous factors may offer a promising strategy to restore muscle mass and function under such pathological states [38]. In this study, we demonstrate that *rh*KAI1 promotes myogenic differentiation in both C2C12 cells and primary HESCs. Treatment with *rh*KAI1 significantly upregulated key myogenic markers, including MyoD and MyHC, and enhanced myotube formation. These effects were accompanied by increased phosphorylation of Akt and AMPK, whereas other regulators such as mTOR, STAT3, and GSK3β remained unchanged. These data suggest that *rh*KAI1-induced myogenesis is associated with modulation of the Akt/AMPK axis, a pathway well documented to support mitochondrial biogenesis, metabolic activation, and myotube maturation. Previous studies have highlighted the role of endogenous CD82/KAI1 in muscle stem cell activation and membrane repair within dystrophic muscle contexts [20,27]. Hall et al. [27] demonstrated that KAI1 is essential for satellite cell activation and muscle regeneration in Duchenne muscular dystrophy models. Similarly, Fontelonga et al. [20] reported that KAI1 interacts with dysferlin and myoferlin, facilitating membrane repair in muscle cells. In contrast, our findings reveal that *rh*KAI1 directly promotes myogenic differentiation in non-dystrophic conditions, suggesting an expanded functional role for KAI1 beyond dystrophic muscle repair. These findings suggest a potential role for *rh*KAI1 in promoting myogenic differentiation beyond previously reported functions related to muscle structural maintenance. Notably, unlike the genetic deficiency models explored previously, we employed a recombinant protein-based approach that may offer potential translational relevance.

Glucocorticoids such as Dex are commonly prescribed for their potent anti-inflammatory and immunosuppressive properties [39]. However, prolonged or high-dose Dex administration has been shown to induce skeletal muscle atrophy, a condition marked by enhanced proteolysis, suppressed protein synthesis, and mitochondrial dysfunction [40]. Mechanistically, this occurs through glucocorticoid receptor-dependent transcriptional activation of muscle-specific E3 ubiquitin ligases, such as Atrogin-1 and MuRF1, which mediate proteasome-dependent degradation of structural muscle proteins [41,42]. This activation upregulates muscle-specific E3 ubiquitin ligases, including atrogin-1 and MuRF1, which are key components of the ubiquitin–proteasome system responsible for muscle protein degradation [41,42]. Consistent with this, our findings demonstrated that dexamethasone exposure in C2C12 myotubes led to a significant reduction in fiber diameter and increased expression of these atrogenes, reflecting a robust catabolic response. These changes were accompanied by reduced expression of myogenic markers, including MyoD and MyHC, suggesting impaired myogenic differentiation under Dex exposure. Importantly, treatment with *rh*KAI1 attenuated several of these atrophic changes, including the preservation of fiber morphology and suppression of atrogin-1 and MuRF1 expression. These findings align with previous reports showing that FoxO transcription factors, activated under glucocorticoid conditions, suppress myogenic programs while promoting protein degradation pathways [43]. Notably, *rh*KAI1 pretreatment significantly attenuated these atrophic responses by preserving fiber morphology, reducing atrogene expression, and restoring myogenic marker expression. These improvements were also accompanied by increased phosphorylation of Akt and AMPK, suggesting that these pathways may be associated with the observed protective effects. It has been well established that Akt inhibits FoxO-mediated transcription of catabolic genes, thereby preventing muscle protein degradation, while AMPK modulates FoxO activity to promote muscle cell survival and differentiation [44,45]. These results collectively support the conclusion that *rh*KAI1 functions as a potent modulator of glucocorticoid-induced atrophy, acting through coordinated regulation of key anabolic and catabolic signaling pathways.

Glucocorticoid-induced muscle atrophy in vivo has been widely modeled through systemic administration of dexamethasone, which recapitulates key clinical features of steroid-induced myopathy, including body weight loss, muscle mass reduction, and functional impairment [46,47]. This model is widely used for evaluating potential therapeutic candidates, as it exhibits characteristic molecular alterations such as elevated expression of muscle-specific E3 ubiquitin ligases and disruption of myofiber composition [5,48]. Numerous studies have reported that the overexpression of these ligases contributes to muscle mass loss under glucocorticoid exposure [5,46,47,48]. Dexamethasone-treated mice exhibited a decrease in body weight, which is commonly observed in glucocorticoid-induced myopathy models and reflects systemic catabolic stress [5,43]. This outcome aligns with previous reports showing that corticosteroid exposure disrupts energy balance and promotes proteolysis in both lean and skeletal muscle compartments [49]. Interestingly, *rh*KAI1 treatment attenuated this weight loss, suggesting a potential effect on systemic metabolic responses. Given that body weight is a global indicator rather than a muscle-specific measure, this improvement may reflect broader systemic effects of *rh*KAI1. Although individual hindlimb muscle weight did not differ significantly across groups, we observed a consistent trend toward increased mass in *rh*KAI1-treated mice. Similar findings were reported in Rutin-treated Dex models, where muscle weight improvements were evident despite subtle statistical shifts depending on the treatment duration and sensitivity of measurement endpoints [43]. Although *rh*KAI1 treatment showed a trend toward increased muscle mass, these changes did not reach statistical significance when assessed by gross weight measurement. Future studies incorporating more sensitive approaches, such as imaging-based volumetric analysis, may help further clarify potential structural effects. In parallel with morphological assessments, myofiber integrity at a systemic level was evaluated by measuring serum myoglobin. Elevated myoglobin in the Dex-treated group is indicative of sarcolemmal compromise and myofiber degeneration, findings that have been consistently reported in corticosteroid-induced atrophy models [5]. Importantly, *rh*KAI1 administration reduced serum myoglobin levels toward baseline, suggesting a potential stabilizing effect on muscle membrane integrity. This finding complements our histological data, suggesting that *rh*KAI1 may influence muscle integrity at both cellular and systemic levels. To assess neuromuscular function, grip strength and rotarod performance tests were conducted, which are standard behavioral assays for quantifying muscle force and coordination in rodent models of muscle atrophy. Dexamethasone-treated animals exhibited significant impairments in both parameters, consistent with previous studies demonstrating that glucocorticoids reduce voluntary locomotion, endurance, and muscle force production. These observations are consistent with prior findings that glucocorticoids impair neuromuscular coordination, endurance, and voluntary movement in rodent models [10,50]. Our present findings show that *rh*KAI1 administration significantly improved both performance metrics compared with the Dex-treated group, indicating partial recovery of neuromuscular function consistent with previous reports in muscle atrophy models [5,10,50]. Consistent with systemic biochemical alterations, histological analysis of GA muscle provided additional structural evidence of Dex-induced muscle atrophy. As previously described in glucocorticoid-treated models, we observed significant reductions in muscle fiber diameter and overall architectural integrity [51,52]. Interestingly, *rh*KAI1-treated mice demonstrated partial recovery of myofiber size and organization, suggesting a potential effect on tissue morphology under catabolic stress. *rh*KAI1 attenuated these changes, as evidenced by improved fiber architecture and reduced expression of atrogenes at the tissue level. These observations suggest that *rh*KAI1 may influence both structural and molecular alterations associated with Dex-induced muscle atrophy. At the molecular level, we further observed clear evidence of fiber type remodeling. Our findings show that Dex exposure increased the expression of MYH4, a marker of fast-twitch glycolytic type IIb fibers, while decreasing MYH7, indicative of oxidative type I fibers. This pattern is characteristic of a slow-to-fast fiber-type transition observed in glucocorticoid-induced muscle atrophy models, and is associated with repression of mitochondrial regulators such as PGC-1α [6,53]. *rh*KAI1 partially reversed this dysregulation by increasing MYH7 expression and reducing MYH4 levels, suggesting a potential role in modulating fiber-type composition under catabolic challenge. This observation is consistent with previous reports indicating that glucocorticoids promote fiber-type switching from oxidative to glycolytic profiles [6,40,53,54]. This transition is mediated through downregulation of mitochondrial regulators such as PGC-1α and increased activity of FoxO transcription factors, both of which are key modulators of metabolic adaptation and fiber identity [40]. Thus, *rh*KAI1 may be associated with modulation of transcriptional regulators involved in oxidative metabolism and fiber identity. These findings suggest that *rh*KAI1 may contribute to maintaining fiber-type distribution, potentially through pathways related to oxidative metabolism.

## 4. Materials and Methods

### 4.1. Preparation of rhKAI1 Protein

*rh*KAI1 protein (catalog No. 12275-H08H) was purchased from Sino Biological Inc. (Beijing, China) as previously described [26]. A DNA sequence encoding the second extracellular domain of human KAI1 (P27701-1) (Gly111-Leu228) was fused with a poly-histidine tag at the C-terminus and a signal peptide at the N-terminus.

### 4.2. C2C12 Cell Culture and Myotube Differentiation

Mouse myoblast C2C12 cells were purchased from the American Type Culture Collection (ATCC; Manassas, MD, USA). The cells were maintained in Dulbecco’s Modified Eagle’s Medium (DMEM, Hyclone, Logan, UT, USA) supplemented with 10% fetal bovine serum (FBS; Invitrogen-Gibco, Carlsbad, CA, USA) and 1% penicillin/streptomycin (Invitrogen-Gibco) at 37 °C in a humidified 5% CO_2_ incubator. For myotube differentiation, confluent cells were exposed to a differentiation medium consisting of DMEM supplemented with 2% heat-inactivated horse serum (Invitrogen-Gibco) and various concentrations of *rh*KAI1 (0, 200, 400, and 800 ng/mL). The differentiation medium was refreshed every 2 days for a total duration of 6 days (Figure 1A).

### 4.3. Primary Human Endometrial Stromal Cells (HESCs) Culture and Myogenic Differentiation

Primary HESCs (passage 1) were isolated from a mid-secretory phase donor’s endometrial biopsy and were kindly provided by Prof. In-Sun Hong at Gachon University. This study was approved by the Institutional Review Board of the Gachon University Gil Medical Center (IRB No. GBIRB2018-134). HESCs were maintained in DMEM/Ham’s F-12 medium (DMEM/F-12, 1:1 mixture) with 3.1 g/L glucose, 1 mM sodium pyruvate, and without phenol red (Sigma-Aldrich Chemical Co., St. Louis, MO, USA), supplemented with 1.5 g/L sodium bicarbonate, 1% ITS+ Premix (BD Biosciences, Bedford, MA, USA), 500 ng/mL puromycin, and 10% charcoal/dextran-treated FBS (Hyclone). To assess the effect of *rh*KAI1 on the myogenic differentiation of HESCs, cells were treated with DMEM/F-12 containing 10% FBS, supplemented with or without 400 ng/mL *rh*KAI1 for 2 weeks (Figure 2A).

### 4.4. Giemsa Staining

Differentiated C2C12 cells and HESCs were gently washed three times with PBS and fixed in 4% paraformaldehyde for 10 min, followed by staining with a 10% Giemsa staining solution (Sigma-Aldrich) for 10 min. The cells were then washed twice with distilled water and visualized using an inverted phase-contrast microscope (Olympus, Tokyo, Japan).

### 4.5. Quantitative Real Time (qRT)-Polymerase Chain Reaction (PCR) Analysis

Total RNA was extracted using Tri-RNA Reagent (FAVORGEN, Ping-Tung, Taiwan) and the cDNA was synthesized using a cDNA synthesis kit (AccuPower^®^, Bioneer, Daejeon, Republic of Korea) in accordance with the manufacturer’s instructions. qPCR was carried out utilizing the SYBR Premix Ex Taq Kit (Takara Biotechnology, Otsu, Japan) and amplified by the ABI QuantStudio3 (Applied Biosystems, Carlsbad, CA, USA). All primers used in this study are listed in the Supplementary Table S1.

### 4.6. Immunofluorescence Staining for MyHC and MyoD

C2C12 myotube differentiation was performed as previously described with slight modifications [26]. Cells were fixed in 4% paraformaldehyde, permeabilized using 0.2% Triton X-100, and blocked in 3% bovine serum albumin in phosphate-buffered saline (PBS) for 30 min at room temperature. The cells were then incubated with MYHC (catalog No. sc-376157, Santa Cruz Biotechnology, Santa Cruz, CA, USA) and MyoD (catalog No. sc-760, Santa Cruz Biotechnology) antibodies for 3 h. This was followed by incubation with Alexa Fluor™ 488-labeled goat anti-mouse (catalog No. A11001, Invitrogen, Carlsbad, CA, USA) and Alexa Fluor™ 647-labeled goat anti-rabbit (catalog No. A21244, Invitrogen) secondary antibodies for 30 min. The cells were then incubated with 4′,6′-diamidino-2-phenylindole (DAPI; Sigma-Aldrich Chemical Co.) for 10 min to visualize nuclei. Immunofluorescence images were obtained using a confocal laser scanning microscope (Carl Zeiss, Oberkochen, Germany).

### 4.7. In Vitro Model of Dexamethasone-Induced Muscle Atrophy

C2C12 myoblasts were differentiated into myotubes in DMEM containing 2% heat-inactivated horse serum for 6 days. After 6 days of differentiation, the myotubes were pretreated with 400 ng/mL *rh*KAI1 for 3 h and then exposed to 10 μM dexamethasone (Sigma-Aldrich) for 24 h (Figure 3A) [55].

### 4.8. Animal Model of Dexamethasone-Induced Muscle Atrophy

All animal care procedures undertaken in this study were approved by the Pusan National University Yangsan Hospital Institutional Animal Care and Use Committee (PNUYH-IACUC approval no. LT2024-002-A1C0). Animal housing and care were conducted at the Pusan National University Yangsan Hospital Biomedical Research Institute under specific pathogen-free conditions. Eighteen C57BL/6N (10-week-old male) mice were purchased from Samtako Bio Korea Co. (Osan, Republic of Korea) and were housed in a controlled environment (22 ± 2 °C temperature, 45 ± 10% humidity, 12/12 h light/dark cycle) where they were acclimated for one week prior to experimentation. After the one-week acclimation, all mice were subjected to behavioral tests for baseline correction. Twelve mice were administered intraperitoneal (i.p.) injections of 20 mg/kg dexamethasone daily for 10 days to induce muscle atrophy, as described previously [43]. After 10 days of dexamethasone treatment, the mice were randomly divided into three groups: Dex (i.p. injection of normal saline, *n* = 4), K200 (i.p. injection of 200 μg *rh*KAI1/kg body weight, *n* = 4), and K400 (i.p. injection of 400 μg *rh*KAI1/kg body weight, *n* = 4). Normal saline and *rh*KAI1 were administered via i.p. injection every three days for 10 days. Non-treated normal mice (control group, *n* = 6) received normal saline injections during the same period (Figure 4A).

### 4.9. Grip Strength and Rotarod Test

At days 0, 10, and 20, all mice were subjected to behavioral tests. As previously described, grip strength, which reflects muscle strength, was measured using a grip strength meter (Jeung Do Bio & Plant, Seoul, Republic of Korea) to evaluate the muscle strength of the mice [56]. In brief, each mouse was positioned to grasp the wire grid, and the tail end was gently pulled backward. Peak grip strength was recorded automatically when the mouse released its grip on the wire grid. This procedure was repeated three times for each animal examined, and the resulting average value was subjected to statistical analysis.

To assess muscle endurance of mice, the rotarod test was conducted using a Mouse Rota-Rod (B.S. Technolab, Seoul, Republic of Korea), following previously described protocols with some modifications [57]. After acclimatizing the mice to the fixed rod, the motor coordination of each individual mouse was determined at 40 rpm for 180 s. Each mouse underwent three rotarod trials, and the duration of time that the animals remained on the rod was recorded. The total time and distance were calculated as the mean value.

### 4.10. Collection of Serum and Muscle Tissues

On the last day of the experiment, all animals were euthanized, and whole blood was collected from the heart. Serum was collected by centrifugation of blood samples at 1200× *g* for 10 min at 4 °C and stored at −80 °C for subsequent analysis. Serum biochemical profiles were analyzed using a Cobas 8000 C702 chemistry analyzer (Roche, Mannheim, Germany). Serum myoglobin levels were measured using a Mouse Myoglobin MYO/MB ELISA kit (catalog No. EK730264, AFG Scientific, Northbrook, IL, USA) according to the manufacturer’s instructions. Skeletal muscle tissues, including the GA, tibialis anterior, soleus, quadriceps, and extensor digitorum longus muscles, were excised from the hindlimbs. Subsequently, isolated muscle tissues were weighed and photographed. GA tissues were immediately stored at −80 °C for Western blot analysis, and part of the GA tissue was embedded in Tissue-Tek OCT compound and then stored at −80 °C until used for histological analysis.

### 4.11. Histological Analysis of GA Muscles

Frozen tissue sections were prepared at a thickness of 10 μm using a cryostat (Leica CM1900, Leica, Wetzlar, Germany) at −25 °C to −30 °C. For hematoxylin and eosin (H&E) staining, individual tissue sections were stained with H&E solution (Sigma-Aldrich), as previously described [58]. Myofiber size was quantified by measuring the minimal Feret diameter using ImageJ^®^ software version 1.53 (National Institutes of Health, Bethesda, MD, USA). Approximately 90 myofibers per group were randomly selected from multiple fields for analysis, and the mean value per animal was used for statistical evaluation. Immunofluorescence staining for MyHC, Atrogin-1, MYH4, and MYH7 was conducted as previously described [59]. The sections were blocked with 2% horse serum for 1 h at room temperature, followed by overnight incubation with primary antibodies at 4 °C and 1 h incubation with secondary antibodies at room temperature. All antibodies used in this study are listed in the Supplementary Table S2. Images were acquired using an Axio Scan Z1 digital fluorescence slide scanner (Carl Zeiss AG, Oberkochen, Germany). Mean fluorescence intensity was quantified using ImageJ^®^ version 1.53 from multiple random fields per condition.

### 4.12. Western Blot Analysis

Total protein from cells and GA muscles were extracted as previously described [43]. Equal amounts of protein were loaded, separated by sodium dodecyl sulfate-polyacrylamide gel electrophoresis (SDS-PAGE), and transferred onto polyvinylidene difluoride membranes. The membranes were blocked with 5% skim milk in Tris-buffered saline containing 0.1% Tween-20, subsequently probed with primary antibodies overnight at 4 °C, and then incubated with the corresponding secondary antibodies for 1 h at room temperature. All antibodies used in this study are listed in the Supplementary Table S3. The membranes were then exposed to an enhanced chemiluminescence solution (Thermo Fisher Scientific, Waltham, MA, USA) and visualized using a LAS-3000 Imaging System (Fujifilm Image Reader, Valhalla, NY, USA).

### 4.13. Statistical Analysis

All data are expressed as the mean ± standard deviation. Statistical analysis was performed using GraphPad Prism 8.0.2 (GraphPad Software, Inc., San Diego, CA, USA), with one-way analysis of variance (ANOVA) for multiple comparisons, followed by Tukey’s post hoc test. Statistical significance was set at *p* values of <0.05 all analysis.

## 5. Conclusions

This study demonstrates that *rh*KAI1 alleviates several features associated with glucocorticoid-induced skeletal muscle atrophy. Using both in vitro and in vivo models, we show that *rh*KAI1 improves several structural and molecular alterations, including myotube morphology and atrogene expression. Furthermore, *rh*KAI1 was associated with increased phosphorylation of Akt and AMPK and improved neuromuscular performance impaired by dexamethasone exposure. Notably, *rh*KAI1 modulated fiber-type-related gene expression associated with dexamethasone exposure. These findings suggest that *rh*KAI1 may represent a candidate molecule for further investigation in steroid-induced muscle dysfunction.

## Data Availability

The original contributions presented in this study are included in the article/Supplementary Materials. Further inquiries can be directed to the corresponding authors.

## Supplementary Materials

The following supporting information can be downloaded at: https://www.mdpi.com/article/10.3390/ijms27062555/s1.

## Abbreviations

The following abbreviations are used in this manuscript:

*rh*KAI1	Recombinant human KAI1/CD82
Dex	Dexamethasone
HESCs	Human endometrial stromal cells
MyHC	Myosin heavy chain
MyoD	Myogenic differentiation 1
Myf5	Myogenic factor 5
AMPK	AMP-activated protein kinase
mTOR	Mechanistic target of rapamycin
FoxO	Forkhead box O transcription factor
MuRF1	Muscle RING-finger protein 1
H&E	Hematoxylin and eosin
i.p.	Intraperitoneal
