# Supplementary material for: Recombinant Human KAI1/CD82 Attenuates Glucocorticoid-Induced Muscle Atrophy by Promoting Myogenic Differentiation
Source: Int J Mol Sci. 2026 Mar 11;27(6):2555. doi: 10.3390/ijms27062555 (PMC13026986; doi:10.3390/ijms27062555)
Supplement: Supplementary file 1 [file ijms-27-02555-s001.zip › ijms-4161903-supplementary.pdf]

Supplementary Table S1. Information of primers used for qPCR analysis.

Gene	Primer Sequence (5'-3')	
	Forward	Reverse
mMyf5	GGTGGAGAACTATTACAGCCTGC	ACAGTAGATGCTGTCAAAGCTGC
mMyoD	GCCTGAGCAAAGTGAATGAG	CGCTACATCGAAGGTCTGC
mMuRF1	TACCAAGCCTGTGGTCATCCTG	ACGGAAACGACCTCCAGACATG
mMyogenin	GAGCGCGATCTCCGCTACAGAGG	CTGGCTTGTGGCAGCCCAGG
mMyHC	CTCTGGCTTACCTCTTCTCTG	TAGAGGATCCTGCTGGGGAA
mAtrogin-1	CTTCTCGACTGCCATCCTGGAT	TCTTTTGGGCGATGCCACTCAG
hPAX7	GGAGGATGAAGCGGACAAGAAG	AGGTCAGGTTCCGACTCCACAT
hMYF5	CAGTCCTGTCTGGTCCAGAAAG	GTCCACTATGTTGGATAAGCAATC
hDYSTROPHIN	GCTCAACCATCGTTTGCAGCC	TTCAGCCTCCAGTGGTTCAAGC
hMYOGENIN	AGTGCCATCCAGTACATCGAGC	AGGCGCTGTGAGAGCTGCATTC

mMyf5, mouse myogenic factor5; *mMyoD*, mouse myoblast determination protein 1; *mMuRF1*, mouse muscle RING-finger protein-1; *mMyHC*, mouse myosin heavy chain; *hPAX7*, human paired box 7.

Supplementary Table S2. Primary and secondary antibodies used for immunofluorescence

Antibodies	Supplier	Catalog No.	Dilution
MyHC	Santa Cruz Biotechnology, Inc	sc-376157	1:500
Atrogin-1	Bioss	Bsm-54451R	1:500
MYH4	Invitrogen	53-6503-82	1:500
MYH7	Santa Cruz Biotechnology, Inc	sc-53089	1:500
Alexa Fluor^{TM} 488-labeled goat anti-mouse	Invitrogen	A11001	1:1,000
Alexa Fluor^{TM} 647-labeled goat anti-rabbit	Invitrogen	A21244	1:1,000

MyHC, myosin heavy chain; MYH4, myosin-4; MYH7, myosin-7. Santa Cruz Biotechnology, Inc. (Santa Cruz, CA, USA); Bioss (Beijing, China); Invitrogen (Waltham, MA, USA)

Supplementary Table S3. Primary and secondary antibodies used for immunoblotting

Antibodies	Supplier	Catalog No.	Dilution
MyHC	Santa Cruz Biotechnology, Inc.	sc-376157	1:1,000
p-Akt	Cell Signaling Technology	4060S	1:1,000
t-Akt	Cell Signaling Technology	9272S	1:1,000
p-AMPK	Cell Signaling Technology	50081S	1:1,000
t-AMPK	Cell Signaling Technology	5831S	1:1,000
p-mTOR	Cell Signaling Technology	5536S	1:1,000
t-mTOR	Cell Signaling Technology	2983S	1:1,000
p-STAT3	Cell Signaling Technology	9145S	1:1,000
t-STAT3	Cell Signaling Technology	4904P	1:1,000
p-GSK3β	Cell Signaling Technology	5558S	1:1,000
t-GSK3β	Cell Signaling Technology	12456S	1:1,000
β-actin	Proteintech Group, Inc.	66009-1	1:5,000
goat anti-mouse IgG-HRP	Santa Cruz Biotechnology, Inc.	sc-2005	1:2,500
goat anti-rabbit IgG-HRP	Santa Cruz Biotechnology, Inc.	sc-2004	1:2,500

Santa Cruz Biotechnology, Inc. (Santa Cruz, CA, USA); Cell Signaling Technology (Beverly, MA, USA); Proteintech Group, Inc. (Rosemont, IL, USA).
